# Ocular and Clinical Characteristics Associated with the Extent of Posterior Lamina Cribrosa Curve in Normal Tension Glaucoma

**DOI:** 10.1038/s41598-018-19321-1

**Published:** 2018-01-17

**Authors:** Seung Hyen Lee, Tae-Woo Kim, Eun Ji Lee, Michaël J. A. Girard, Jean Martial Mari, Robert Ritch

**Affiliations:** 1 Department of Ophthalmology, Bundang Jesaeng General Hospital, Daejin Medical Center, Seongnam, Korea; 2 Department of Ophthalmology, Seoul National University College of Medicine, Seoul National University Bundang Hospital, Seongnam, Korea; 3 Department of Biomedical Engineering, National University of Singapore, Singapore, Singapore; 4 Singapore Eye Research Institute, Singapore National Eye Centre, Singapore, Singapore; 5 Université de la Polynésie française, Tahiti, French Polynesia; 6 Einhorn Clinical Research Center, New York Eye and Ear Infirmary of Mount Siani, New York, New York, United States

## Abstract

Although normal-tension glaucoma (NTG) is pathogenetically heterogenous, there have been few attempts to subclassify NTG patients according to the mechanism and anatomy of optic nerve damage. This cross-sectional study was performed to investigate differences in the clinical and ocular characteristics between NTG patient groups stratified according to the degree of posterior lamina cribrosa (LC) curve which was assessed by calculating LC curvature index (LCCI). A total of 101 eyes of 101 treatment naïve NTG patients were included. The optic nerve head was imaged using enhanced-depth-imaging spectral-domain optical coherence tomography in three horizontal B-scan images in each eye. The patients were divided into two groups based on the magnitude of LCCI using a cutoff of known upper 95 percentile value in healthy subjects: a steeply curved LC group (Group 1, 75 eyes, 74.3%) and a relatively flat LC group (Group 2, 26 eyes, 25.7%). NTG eyes with relatively flat LC had lower intraocular pressure, and were associated with greater parapapillary structural alternation and systemic risk factors. These data suggest that assessment of LC morphology may help clinicians seek additional risk factors and make inferences about the mechanism of optic nerve damage in individual patients.

## Introduction

Glaucoma is a progressive optic neuropathy characterized by a typical pattern of optic nerve damage and visual field loss. Although elevated intraocular pressure (IOP) is the most important known risk factor for glaucoma onset and progression^1,2^, other factors, including vascular disorders, are considered to contribute to glaucomatous optic nerve damage individually or collectively^3,4^. The role of vascular factors may be particularly important in eyes with glaucoma occurring at what are considered normal IOP (normal-tension glaucoma). These vascular risk factors may in themselves be considered diseases, such as peripheral vascular dysfunction, low nocturnal blood pressure, and obstructive sleep apnea. In addition, a group of risk factors is related to neurodegenerative disorders in general, such as mitochondrial dysfunction, oxidative damage, and low-grade inflammation. Currently and historically, however, treatment has been targeted only to lowering IOP in both normal-tension glaucoma and high-tension glaucoma. If patients can be stratified according to their predominant pathogenic mechanism (e.g, predominant involvement of IOP-related factors vs. non-IOP related factors), clinicians might be more able to provide a more specific targeted treatment approach to individual patients.

The lamina cribrosa (LC) is a mesh-like structure which provides structural and functional support to the retinal ganglion cell (RGC) axons. It has been considered that posterior displacement, compression, and remodeling of the LC driven by the translaminar pressure difference (TLPD) are the principal pathogenic events^5–7^. The LC deformation and/or remodeling can cause blockade of axonal transport, which would ultimately lead to RGC apoptosis. Experimental studies have supported this concept by demonstrating posterior bowing of the LC after IOP elevation in an early glaucoma model^8,9^.

Given that LC deformation (posterior bowing) is the net result of IOP-induced stress and opposing factors (e.g., retrolaminar tissue pressure or resistance of the LC), it can be proposed that the extent (i.e.,steepness) of LC curve might be useful as an index to evaluate the relative importance of translaminar stress in a given patient. For instance, it may be presumed that translaminar stress/strain is largely involved in eyes with steep LC curve regardless of the level of IOP. Low retrolaminar tissue pressure^10^ or an unusually flexible LC^5^ may be an associated factor in such a case. On the other hand, other factors may play a greater role in eyes with relatively flat LC curve, even if their IOP is relatively high. However, there is a lack of evidence supporting this idea.

It can be postulated that the pathogenesis of any two diseases are different when they have considerable differences in demographic or associated clinical features. Based on this idea, we compared the clinical and ocular characteristics between NTG patient groups stratified according to the magnitude of LC curve.

## Results

### Baseline Characteristics

One hundred eighteen treatment-naïve NTG patients were initially included. Of these, 17 were excluded due to poor scan image quality (>5 missing sections in an average of 75 sections). The LC, especially its anterior surface, was readily discernible in most of the B-scan images at examinations in the remaining 101 patients.

**Figure 1:**
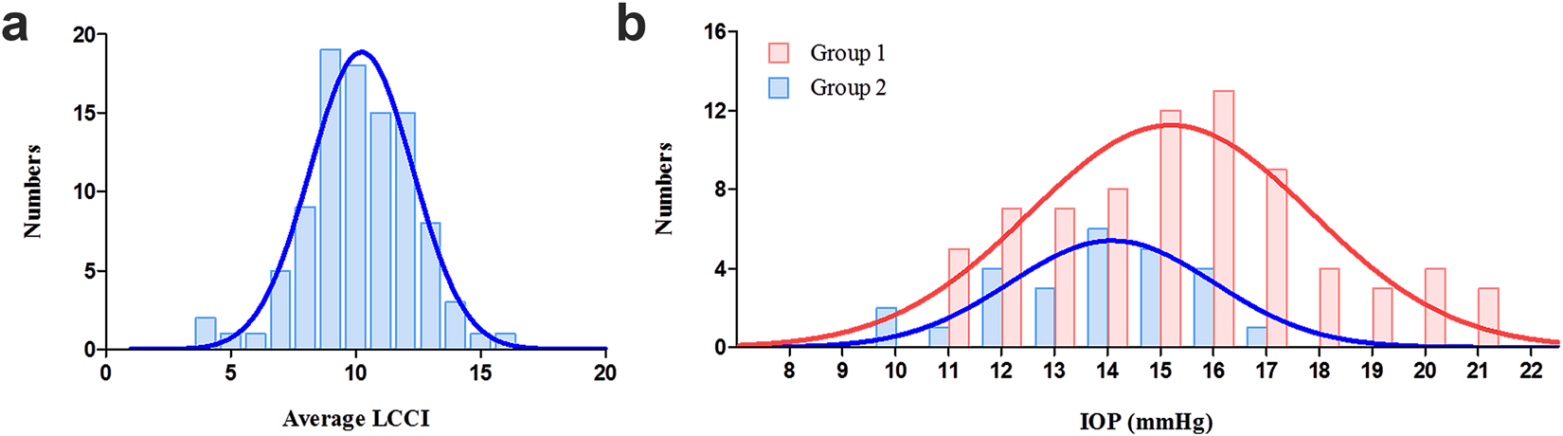
Histogram showing the distribution of average LCCI and intraocular pressure (IOP) in naïve normal-tension glaucoma (*n* = 101). (**a**) The LCCI distribution showed a normal curve (Gaussian curve) (*P* = 0.200 by Kolmogorov-Smirnov test). (**b**) The distribution of IOP in both groups. Note that there is a large overlap in the distribution range of IOP between the 2 groups.

Table 1 shows the demographic and clinical characteristics of included subjects. The patients were 57.3 ± 11.9 years old (range, 31–83 years), and 43 (42.6%) were women. The entire cohort had a visual acuity ranging from 20/40 to 20/20, a refractive error (spherical equivalent) of −1.12 ± 2.60 diopters (range, −6.25 to +3.5 diopters), and a visual field mean deviation of −7.59 ± 7.01 dB (range, −27.65 to 0.55 dB). The IOP was 14.7 ± 2.6 mmHg (range, 10–21 mmHg). The mean lamina cribrosa curvature index (LCCI) was 10.8 ± 2.0. The mean frequency distribution of the LCCI showed a Gaussian curve (*P* = 0.200, Fig. 1a) ranging from 5.85 to 16.76.

**Table 1:** Demographic characteristics of the study subjects.

Variables	POAG (n = 101), mean ± standard deviation
Demographic characteristics	
Age (years)	57.3 ± 11.9
Female (%)	43 (42.6)
Clinical characteristics	
Diabetes (%)	14 (13.9)
Hypertension (%)	31 (30.7)
SBP (mmHg)	127.9 ± 14.8
DBP (mmHg)	78.0 ± 11.4
SPP (mmHg)	113.2 ± 14.1
DPP(mmHg)	63.2 ± 11.1
MAP (mmHg)	94.6 ± 11.4
MPP (mmHg)	48.3 ± 7.3
Ocular characteristics	
Baseline IOP (mmHg)	14.7 ± 2.6
Scan IOP (mmHg)	14.9 ± 2.5
SE (D)	−1.12 ± 2.60
AXL (mm)	24.28 ± 1.50
CCT (μm)	553.0 ± 39.5
VF MD (dB)	−7.59 ± 7.01
VF PSD (dB)	6.90 ± 4.19
Global RNFL thickness (μm)	73.2 ± 14.7
β-PPA width (μm)	255.7 ± 147.8
γ-PPA width (μm)	64.5 ± 127.6
JPCT (μm)	141.8 ± 62.1
LCCI	10.80 ± 2.00

POAG, primary open-angle glaucoma; SBP, systolic blood pressure; DBP, diastolic blood pressure; SPP, systolic perfusion pressure; DPP, diastolic perfusion pressure; MAP, mean arterial pressure; MPP = mean perfusion pressure; IOP = intraocular pressure; Scan IOP = IOP at the time of optic nerve head scan; SE = spherical equivalent; D = diopter; AXL = axial length; CCT = central corneal thickness; VF = visual field; MD = mean deviation; dB = decibel; PSD = pattern standard deviation; RNFL = retinal nerve fiber layer; PPA = parapapillary atrophy; JPCT = juxtapapillary choroidal thickness; LCCI = lamina cribrosa curvature index.

Data are mean ± standard deviation or *n* (%) values.

The 95% Bland-Altman limit for interobserver agreement between the measurements from the 2 observers were –0.97 to +1.14 for the LCCI, −3.31 to +2.89 for the juxtapapillary choroidal thickness (JPCT) and −5.23 to +4.91 for the width of parapapillary atrophy (PPA) with Bruch’s membrane (β-PPA).

### Comparison of Clinical and Ocular Characteristics between Groups

There was no difference in retinal nerve fiber layer (RNFL) thickness and mean deviation of visual field test between Group 1 and 2. Eyes in Group 1 were associated with older age (*P* = 0.031), a higher baseline IOP (*P* = 0.033), a larger width of β-PPA and smaller JPCT (*P* = 0.44 and *P* < 0.001, respectively) compared to Group 2. The IOP of eyes in Group 2 ranged from 10 to 17 mmHg, those in Group 1 ranged from 11 to 21 mmHg (Fig. 1b). The SBP, DBP, SPP and DPP were significantly lower in Group 1 than in Group 2 (Table 2, all *P* ≤ 0.021).

**Table 2:** Comparison of Clinical and Ocular Characteristics between Eyes with Steeply curved LC and Relatively flat LC.

Variables	Group 1: Steeply curved LC group (n = 75)	Group 2: Relatively flat LC group (n = 26)	*P* value
Demographic characteristics			
Age (years)	55.8 ± 11.4	61.6 ± 12.3	**0**.**031**
Female (%)	34 (45.3)	9 (34.6)	0.341
Clinical characteristics			
Diabetes mellitus (%)	10 (13.3)	4 (15.4)	0.794
Hypertension (%)	24 (32.0)	7 (26.9)	0.629
Cold extremities (%)	14 (23.0)	7 (38.9)	0.179
SBP (mmHg)	130.1 ± 14.9	121.5 ± 12.7	**0**.**010**
DBP (mmHg)	80.2 ± 11.2	71.5 ± 9.1	**0**.**001**
SPP (mmHg)	115.1 ± 14.4	107.7 ± 12.0	**0**.**021**
DPP(mmHg)	65.1 ± 11.2	57.7 ± 8.8	**0**.**001**
MAP (mmHg)	96.8 ± 11.3	88.2 ± 9.4	**0**.**001**
MPP (mmHg)	49.5 ± 7.4	45.0 ± 5.8	**0**.**006**
Ocular characteristics			
Baseline IOP (mmHg)	15.1 ± 2.7	13.8 ± 1.9	**0**.**033**
Scan IOP (mmHg)	15.3 ± 2.6	13.7 ± 2.0	**0**.**035**
SE (D)	−1.33 ± 2.57	−0.54 ± 2.63	0.184
AXL (mm)	24.36 ± 1.48	24.03 ± 1.57	0.373
CCT (μm)	552.6 ± 40.0	553.9 ± 38.9	0.895
VF MD (dB)	−7.66 ± 7.27	−7.40 ± 6.35	0.876
VF PSD (dB)	6.81 ± 4.12	7.16 ± 4.46	0.725
Global RNFL thickness (μm)	72.1 ± 15.3	76.4 ± 12.4	0.205
β-PPA width (μm)	238.4 ± 151.4	305.8 ± 126.5	**0**.**044**
γ-PPA width (μm)	61.0 ± 113.4	74.5 ± 162.2	0.644
JPCT (μm)	154.5 ± 62.9	105.4 ± 42.9	**<0**.**001**
Average LCCI	11.64 ± 1.50	8.36 ± 0.98	**<0**.**001**
Superior midperiphery LCCI	11.89 ± 1.88	8.33 ± 1.44	**<0**.**001**
Midhorizontal LCCI	10.94 ± 2.17	8.08 ± 1.73	**<0**.**001**
Inferior midperiphery LCCI	12.09 ± 2.17	8.67 ± 2.05	**<0**.**001**

LC = lamina cribrosa; SBP = systolic blood pressure; DBP = diastolic blood pressure; SPP = systolic perfusion pressure; DPP = diastolic perfusion pressure; MAP = mean arterial pressure; MPP = mean perfusion pressure; IOP = intraocular pressure; Scan IOP = IOP at the time of optic nerve head scan; SE = spherical equivalent; D = diopter; AXL = axial length; CCT = central corneal thickness; VF = visual field; MD = mean deviation; dB = decibel; PSD = pattern standard deviation; RNFL = retinal nerve fiber layer; PPA = parapapillary atrophy; JPCT = juxtapapillary choroidal thickness, LCCI = lamina cribrosa curvature index.

Data are mean ± standard deviation values, with statistically significant P values in boldface.

Since β-PPA and JPCT are known to be influenced by age, general linear model (analysis of covariance, ANCOVA) was additionally performed to compare the width of β-PPA and JPCT with adjusting for age. The JPCT was significantly larger in Group 1 than in Group 2 at all three planes (all *P* ≤ 0.016 for JPCT; Table 3). Although mean β-PPA width in Group 1 was significant larger than Group 2 before adjusting for age (*P* = 0.044, Table 2), β-PPA width at each location did not show significant differences between groups after adjusting for age (Table 3).

**Table 3:** The width of parapapillary atrophy (PPA) with Bruch’s membrane (BM) and JPCT (juxtapapillary choroidal thickness) adjusted for age at each locations.

	β-PPA width (μm)			JPCT (μm)		
	Group 1	Group 2	*P*	Group 1	Group 2	*P*
Superior midperiphery	226.6 ± 151.6	304.0 ± 133.0	0.107	158.9 ± 66.6	106.2 ± 41.1	**0**.**002**
Mid-horizontal	239.3 ± 158.1	304.1 ± 140.9	0.294	153.3 ± 62.2	104.6 ± 44.7	**0**.**003**
Inferior midperiphery	249.2 ± 162.9	309.3 ± 125.5	0.393	151.2 ± 68.5	105.4 ± 48.5	**0**.**016**
Average	238.4 ± 151.4	305.8 ± 126.5	0.219	154.5 ± 62.9	105.4 ± 42.9	**0**.**003**

PPA = parapapillary atrophy; JPCT = juxtapapillary choroidal thickness.

Data are mean ± standard deviation values, with statistically significant *P* values in boldface.

Bonferroni correction was applied to raw data for measurements in the 3 meridians. Values that were significant after Bonferroni corrections (*P* < 0.017; 0.05/3) are shown in bold.

Analysis of covariance (ANCOVA) adjusted for age.

### Representative Cases

**Figure 2:**
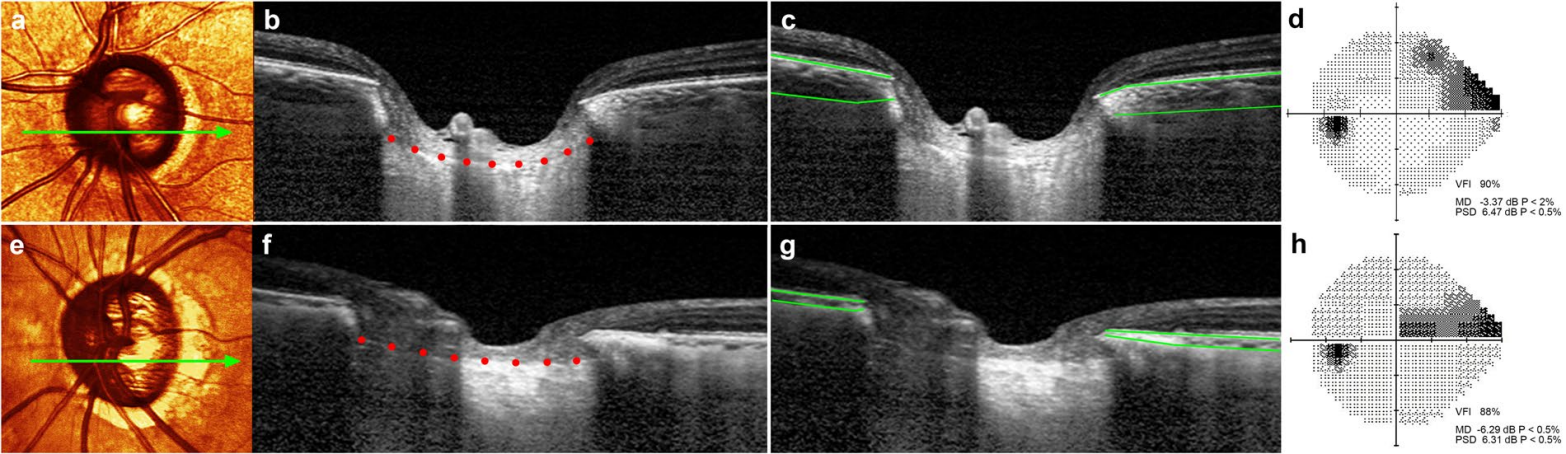
Representative cases showing the relationship between the lamina cribrosa curvature index (LCCI) and ocular characteristics. (**a–d**) Glaucomatous eye of a 57-year-old woman with increased LCCI. (**e–h**) Glaucomatous eye of a 75-year-old man with relatively flat LC curvature. (**a**,**d**) Stereoscopic optic disc photograph images. *Light green arrows* indicate the location of the B-scan. (**b**,**c**) and (**f**,**g**) B-scan images obtained at the locations indicated by *light green arrows* in (**a**) and (**e**), respectively. Note that the LCCI is considerably larger in (**b**) than in (**f**), whereas the JPCT is notably larger in (**c**) than in (**g**). (**d**,**h**) the degree of visual field damage is not largely different between the two eyes. VFI, visual field index; MD, mean deviation; PSD, pattern standard deviation.

Figure 2 shows two eyes, one with a steeply curved LC (Fig. 2a–d) and one with a nearly flat LC (Fig. 2e–h). Considerably smaller JPCT was observed in the latter eye (Fig. 2g) than the former (Fig. 2c), whereas the LCCI is notably larger in (Fig. 2b) than in (Fig. 2f).

## Discussion

The present study demonstrates significant differences in clinical and ocular characteristics between NTG patients stratified according to the LCCI. To our knowledge, this is the first study to relate clinical and ocular characteristics with LC morphology among NTG patients.

The LC morphology was evaluated using LCCI. Previous experimental studies demonstrated that the LC deforms as a posterior bowing pattern when the IOP was elevated^8,9^. Recently, our group demonstrated that LC became less curved after IOP lowering surgery^11^. These findings indicate that the LC curvature is sensitive to the translaminar stress. More recently, our group also demonstrated the LCCI had a capability comparable to the known nerve fiber layer thickness measurement to discriminate glaucoma from healthy eyes^12^. This finding suggests that the LCCI is a valid parameter that characterizes glaucoma-associated LC deformity.

The LCCI was measured at three B-scans. This is based on our observation that the LC curvature is strongly correlated each other within the superior and inferior hemioptic nerve, respectively (Supplementary Video S1)^11,12^. Measuring the LCCI at more planes may be helpful to understand the LC morphology more comprehensively. However, analyzing more planes may have limitations. First, it is difficult to lend the method to the clinical adoption. Analyzing the 3 planes may allow the simple and intuitive assessment of the LC morphology. Second, the superior and inferior midperiphery are the most preferentially damaged areas in glaucoma. If more planes are evaluated and averaged, it may alleviate the assessment of LC bowing.

The patients in Group 2 had lower IOP than those in Group 1. Considering that the IOP is an important component of the translaminar pressure gradient, this finding is to be expected. However, there was a large overlap in IOP between Group 1 and Group 2. This finding is in line with the notion that the connective tissue mechanics within the ONH is not simply governed by the IOP per se but a complex of diverse factors, which may include ocular geometry, material properties, laminar and scleral stiffness or distensibility, and translaminar pressure differential^5,8^. This matter highlights that the ONH damage is attributed to IOP related stress in some NTG patients despite having a statistically normal range of IOP, while non-IOP related factors play a predominant or significant role in addition to IOP related stress in other patients.

The subjects in Group 2 were significantly older than those in Group 1. An influence of age on stiffness of the lamina cribrosa has been demonstrated in several previous studies^13–15^. Collagen type I, III, IV within the cribriform plates increases with age^14,16^. Such alterations may form the biochemical basis for a stiffer property of LC with age. There is a possibility that a stiffer LC with age could serve as a protective mechanism against IOP-induced stress. Based on this consideration, it may be proposed that NTG which develops in old age is relatively more strongly associated with factors other than mechanical stress/strain.

The JPCT was significantly smaller in Group 2. The parapapillary choroid is a vascular layer that lies immediately adjacent to the optic nerve. Since the choroid may be related with the perfusion to the optic nerve, the small JPCT may implicate that the role of vascular factors is relatively greater in eyes with thin choroid. Therefore, the association of thin choroid in Group 2 suggests that optic nerve damage is relatively more attributable to vascular compromise than in Group 1.

Lower perfusion pressure has been a focus of interest as a potential vascular factor for glaucoma. The association of low blood pressure and low perfusion pressure with Group 2 is another finding that implicates that the role of vascular factors is relatively greater in these eyes than in those in Group 1. The difference of clinical characteristics between groups may be ascribed to age difference between groups. However, SBP is known to increase with age^17^. Thus, the lower BP in Group 2 cannot be attributable to age effect.

The β-PPA was greater in Group 2 compared to Group 1. However, the difference was not significant when the age was considered as a covariate. This finding suggests that the apparent larger β-PPA in Group 2 is largely attributable to the older age in Group 2: it is known that the β-PPA is an age-related atrophic change, which enlarges with aging^18,19^. Meanwhile, the β-PPA is characterized histologically by the absence or marked atrophy of the choriocapillaris and choroidal vessels. Such change may facilitate the axonal damage in two ways. First, as described earlier, choroidal derangement may affect the ONH perfusion. Second, atrophy of the choroid may disrupt the blood-optic nerve barrier, thereby potentially allowing the entrance of vasoactive or toxic substance to the ONH from the choroid^20,21^.

Park *et al*.^22^ reported that focal LC defects were observed more in eyes with NTG than in those with HTG. Based on this finding, they postulated that the laminar beams are more likely to be damaged or lost in a localized fashion in NTG, resulting in more localized glaucomatous defects in NTG^23,24^. Our data is in line with their notion that LC deformation occurs in a different fashion depending on the mechanisms involved in the ONH damage, and emphasizes the importance of LC evaluation in glaucoma in addition to conventional assessment. Although the LC evaluation per se cannot determine precisely the specific mechanisms of glaucomatous damage, clinicians may consider workups for non-IOP related risk factors such as sleep apnea^25,26^, nocturnal hypotension^27,28^, and Flammer syndrome^29^ in eyes with relatively flat LC. On the contrary, IOP lowering may initially considered even though the IOP is low when the LC is steeply curved.

This study was subject to limitations. First, we excluded eyes with tilted or torted disc. Thus, current result cannot be applied to those eyes. Second, the criterion of LCCI for the stratification was adapted from our previous study, which included 77 subjects. In addition, a single cutoff value was used for all patients without considering age. A cutoff based on a larger number of healthy subjects and considering the effect of age may be helpful to stratify the POAG patients more accurately.

In conclusion, when stratified based on the degree of posterior LC curve, different characteristics were found between groups. Our data suggest that IOP related stress may relatively play a predominant role in eyes with steeply bowed LC, whereas non-IOP related factors may exert a relatively stronger influence in eyes with less curved LC.

## Methods

The subjects in this study were recruited from an ongoing prospective investigation of glaucoma patients at the Seoul National University Bundang Hospital Glaucoma Clinic: Investigating Glaucoma Progression Study (IGPS)^30,31^, which has been under way since August 2011. This study involves consecutive subjects who met the eligibility criteria and provided written informed consent to participate. It was approved by the Seoul National University Bundang Hospital Institutional Review Board and conformed to the Declaration of Helsinki.

### Study Subjects

Patients included in the IGPS underwent a complete ophthalmic examination including visual acuity assessment, refraction, slit-lamp biomicroscopy, gonioscopy, Goldmann applanation tonometry, and dilated stereoscopic examination of the optic disc, as well as measurement of corneal curvature (KR-1800, Topcon, Tokyo, Japan), central corneal thickness (Orbscan II, Bausch & Lomb Surgical, Rochester, NY), axial length (IOLMaster version 5, Carl Zeiss Meditec, Dublin, CA, USA), stereo disc photography (EOS D60 digital camera, Canon, Utsunomiya-shi, Tochigi-ken, Japan), spectral-domain optical coherence tomography (SD-OCT; Spectralis OCT, Heidelberg Engineering, Heidelberg, Germany), and standard automated perimetry (Humphrey Field Analyzer II 750 24-2 Swedish interactive threshold algorithm; Carl Zeiss Meditec).

Systolic blood pressure (SBP) and diastolic blood pressure (DBP) were measured in the sitting position at the right upper arm with an automated oscillometric device at the time of SD-OCT exam. Ocular systolic perfusion pressure (SPP) was defined as SBP – IOP at the time of SD-OCT exam and ocular diastolic perfusion pressure (DPP) was defined as DBP – IOP. Mean arterial pressure (MAP) was calculated as DBP +1/3(SBP-DBP). Mean ocular perfusion pressure (MPP) was calculated as 2/3 (MAP) – IOP.

The inclusion criteria for the present study were having normal-tension glaucoma (NTG), a best-corrected visual acuity of at least 20/40, a spherical refraction of −6.0 to +3.0 diopters, and a cylinder correction ≤±3.0 diopters. NTG was defined as the presence of glaucomatous optic nerve damage (i.e., the presence of diffuse or localized rim thinning, notching, or a disc hemorrhage), corresponding visual field defect with maximum IOP <22 mmHg (without glaucoma medications), open angle on gonioscopic examination, and no prior history of long term use of steroid medication or no identifiable secondary cause of glaucoma. A glaucomatous visual field change was defined as (1) outside the normal limit on the glaucoma hemifield test, (2) three abnormal points with a *P* < 5% probability of being normal, one point with *P* < 1% by pattern deviation, or (3) a pattern standard deviation of *P* < 5% confirmed on two consecutive reliable tests. A visual field measurement was considered as reliable when both false-positive and false-negative results were ≤25% and fixation losses were ≤20%.

Eyes with a tilted (i.e., defined as a tilt ratio between the longest and shortest diameters of the optic disc of >1.3)^32,33^ or torted disc (i.e., defined as a torsion angle-the deviation of the long axis of the optic disc from the vertical meridian-of >15°)^33,34^, a history of previous intraocular surgery or coexisting retinal (e.g., diabetic retinopathy, retinal vessel occlusion, or retinoschisis) or neurologic diseases (e.g., pituitary tumor) that could affect the visual field were excluded from this study. Secondary glaucoma (e.g., uveitic glaucoma) that may increase IOP was also excluded. Eyes were also excluded when a good-quality image (i.e., quality score >15) could not be obtained in more than five sections. When the quality score did not reach 15, the image-acquisition process automatically stopped, or images of the respective sections were not obtained. Only acceptable scans with a good-quality image that allowed clear delineation of the anterior border of the LC were included.

In cases in which both eyes of a subject were eligible for the study, one eye was selected randomly. Untreated IOP in the NTG patients was defined as the mean value of multiple (typically five) IOP measurements made on the same day (9AM to 5PM) or on different days obtained before IOP-lowering treatment. Optic discs were examined using SD-OCT before IOP lowering medical treatment.

### Enhanced-Depth-Imaging OCT of the Optic Nerve Head

The optic nerve, including the parapapillary area, was imaged using the enhanced-depth-imaging technique of the Spectralis OCT system^35^. This technique yields images with a stronger signal and better image contrast in the deep optic nerve head (ONH) tissue compared to the conventional imaging technique^36^. Patients were imaged through undilated pupils using a rectangle subtending 10 degrees × 15 degrees of the optic disc. This rectangle was scanned with approximately 75 B-scan section images that were separated by 30–34 μm (the scan line distance was determined automatically by the machine). Approximately 42 SD-OCT frames were averaged for each section. This protocol provided the best trade-off between image quality and patient cooperation^36^. Potential magnification errors were avoided by entering the corneal curvature of each eye into the Spectralis OCT system prior to scanning.

### Quantification of LC curvature

**Figure 3:**
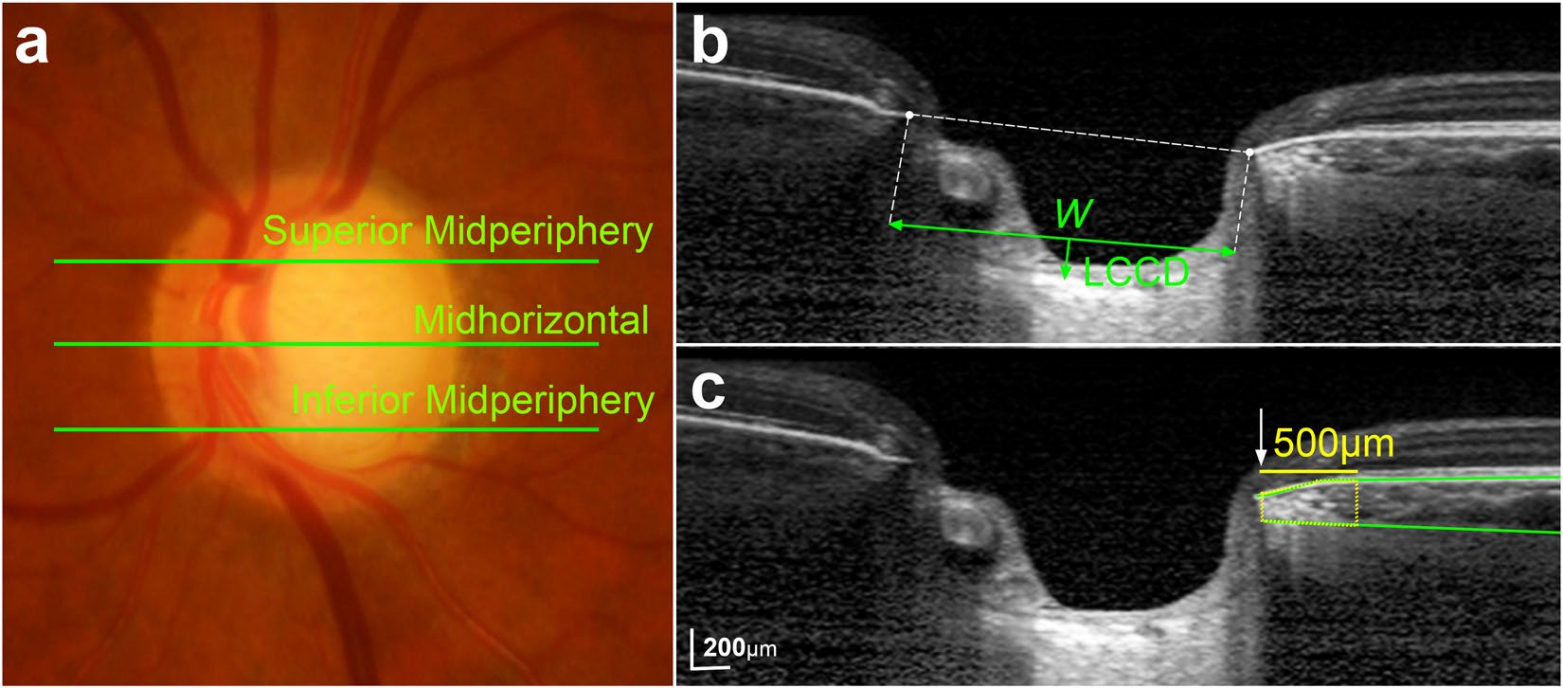
Determination of the lamina cribrosa curvature index (LCCI) and juxtapapillary choroidal thickness (JPCT). (**a**) Sterescopic optic disc photograph image. (**b**,**c**) B-scan images obtained at superior midperiphery as shown in **(a**). (**b**) The LCCI was measured by dividing the lamina cribrosa (LC) curve depth (LCCD) within Bruch’s membrane (BM) opening by length of LC surface reference line (*W*), and then multiplying by 100. (**c**) *Green solid lines* indicate the upper and lower margins of the parapapillary choroid at the superior midperiphery, represented by BM and the choroidoscleral interface, respectively. The area of the juxtapapillary choroidal tissue within 500 μm from the border tissue of Elschnig was measured, and the mean was calculated by dividing the area by 500 μm.

To quantify the posterior LC curve on the SD-OCT B-scan images, we defined the LCCI as the inflection of a curve representing a section of the LC. The measurement of LCCI has been described elsewhere^12^. In brief, a reference line (LC surface reference line) was set in each B-scan by connecting the two points on the anterior LC surface which met with the lines drawn from each Bruch’s membrane termination point. The length of this reference line was defined as width (*W*). The lamina cribrosa curve depth (LCCD) was determined as the maximum depth from this reference line to the anterior surface (Fig. 3). The LCCI was then calculated as (LCCD/*W*) × 100. Since the curvature was thereby normalized according to LC width, LCCI represents the posterior curvature of the anterior LC surface independent of the actual size of the ONH. Only the LC within the Bruch’s membrane opening (BMO) was considered because the LC was often not clearly visible outside of the BMO.

Before the measurement, the visibility of the peripheral LC was enhanced by post processing images using adaptive compensation^37,38^. Measurement was performed using a manual caliper tool in the Amira software (version 5.2.2, Visage Imaging, Berlin, Germany) at three selected B-scan images of the about 75 B-scan section images, spaced equidistant across the vertical optic disc diameter in each eye. These three B-scan lines were defined as superior midperiphery, midhorizontal and inferior midperiphery (Fig. 3), respectively. The LCCIs were measured by two experienced observers (S.H.L. and E.J.L.) who were masked to the clinical information. The average of the measurements from each of the two observers was used for analysis.

Based on the measured LCCI, subjects were divided into two groups using a LCCI of 9.51 as a cutoff value, which was a upper 95 percentile value of normal subjects in our previous study^12^: more steeply curved LC group (Group 1) and relatively flat curved LC group (Group 2).

### Measurement of Juxtapapillary Choroidal Thickness and Parapapapillary Atrophy

The JPCT and the width of the β-zone PPA were measured using a manual caliper tool in the Amira software (version 5.2.2, Visage Imaging, Berlin, Germany), as previous described^39,40^, on same three B-scans that were used in the LCCI measurements. In each B-scan, the area of the choroidal tissue within 500 μm from the border tissue of Elschnig was measured and the JPCT was obtained by dividing the measured choroidal area by 500 μm. The mean JPCT was calculated using the mean of the 3 JPCT measurements obtained from the three B-scans. In eyes with externally or internally oblique border tissue^41,42^, the inner margin of the area of interest was determined at the innermost location at which the perpendicular distance between Bruch’s membrane (BM) and the choroidoscleral interface could be discerned (Fig. 3)^41,43^.

The β-zone PPA was categorized into β-PPA and γ-PPA on the basis of the location of BM termination within the β-zone PPA area, as previously described^44^. The β-PPA was defined as the PPA with BM, and γ-PPA was defined as the PPA without BM. The mean PPA width was calculated by taking the mean of the 3 location measurements (superior midperiphery, midhorizontal, inferior midperiphery). Measurements of JPCT and PPA width were performed by 2 experienced ophthalmologists (S.H.L. and E.J.L.) who were blinded to the subjects’ clinical information. The average of the measurements of the JPCT and PPA from each of the two observers was considered for analysis, respectively.

### Statistical Analysis

The interobserver agreement for measuring the LCCI, JPCT and PPA width were evaluated by calculating the 95% limits of agreement according to Bland-Altman. Between-groups comparisons were performed using the Student *t*-test for continuous variables and the chi-square test for categorical variables. General linear models (ANCOVA) adjusted for age was performed to compare JPCT and β -PPA between groups at each location. All of the statistical analyses were performed using the Statistical Package for the Social Sciences software (version 22.0, SPSS, Chicago, IL, USA). Probability values of *P* < 0.05 were considered indicative of statistical significance. Except where stated otherwise, the data are presented as mean ± standard deviation values.

### Data availability

Data supporting the findings of the current study are available from the corresponding author on reasonable request.

## Electronic supplementary material

Supplementary Video 1

Supplementary Information

## References

[1] The Advanced Glaucoma Intervention Study (AGIS): 7 (2000). The relationship between control of intraocular pressure and visual field deterioration.The AGIS Investigators. Am J Ophthalmol.

[2] Leske MC (2003). Factors for glaucoma progression and the effect of treatment: the early manifest glaucoma trial. Arch Ophthalmol.

[3] Hayreh SS (1969). Blood supply of the optic nerve head and its role in optic atrophy, glaucoma, and oedema of the optic disc. Br J Ophthalmol.

[4] Flammer J, Orgul S (1998). Optic nerve blood-flow abnormalities in glaucoma. Prog Retin Eye Res.

[5] Burgoyne CF, Downs JC, Bellezza AJ, Suh JK, Hart RT (2005). The optic nerve head as a biomechanical structure: a new paradigm for understanding the role of IOP-related stress and strain in the pathophysiology of glaucomatous optic nerve head damage. Prog Retin Eye Res.

[6] Sigal IA, Flanagan JG, Tertinegg I, Ethier CR (2007). Predicted extension, compression and shearing of optic nerve head tissues. Exp Eye Res.

[7] Jonas JB, Berenshtein E, Holbach L (2004). Lamina cribrosa thickness and spatial relationships between intraocular space and cerebrospinal fluid space in highly myopic eyes. Invest Ophthalmol Vis Sci.

[8] Bellezza AJ (2003). Deformation of the lamina cribrosa and anterior scleral canal wall in early experimental glaucoma. Invest Ophthalmol Vis Sci.

[9] Yan DB (1994). Deformation of the lamina cribrosa by elevated intraocular pressure. Br J Ophthalmol.

[10] Jonas JB (2015). Estimated trans-lamina cribrosa pressure difference versus intraocular pressure as biomarker for open-angle glaucoma. The Beijing Eye Study 2011. Acta Ophthalmol.

[11] Lee SH (2016). Reduction of the Lamina Cribrosa Curvature After Trabeculectomy in Glaucoma. Invest Ophthalmol Vis Sci.

[12] Lee SH, Kim TW, Lee EJ, Girard MJ, Mari JM (2017). Diagnostic Power of Lamina Cribrosa Depth and Curvature in Glaucoma. Invest Ophthalmol Vis Sci.

[13] Albon J, Purslow PP, Karwatowski WS, Easty DL (2000). Age related compliance of the lamina cribrosa in human eyes. Br J Ophthalmol.

[14] Hernandez MR, Luo XX, Andrzejewska W, Neufeld AH (1989). Age-related changes in the extracellular matrix of the human optic nerve head. Am J Ophthalmol.

[15] Morrison JC, Jerdan JA, Dorman ME, Quigley HA (1989). Structural proteins of the neonatal and adult lamina cribrosa. Arch Ophthalmol.

[16] Albon J, Karwatowski WS, Avery N, Easty DL, Duance VC (1995). Changes in the collagenous matrix of the aging human lamina cribrosa. Br J Ophthalmol.

[17] Franklin SS (1997). Hemodynamic patterns of age-related changes in blood pressure. The Framingham Heart Study. Circulation.

[18] Curcio CA, Saunders PL, Younger PW, Malek G (2000). Peripapillary chorioretinal atrophy: Bruch’s membrane changes and photoreceptor loss. Ophthalmology.

[19] Spaide RF (2009). Age-related choroidal atrophy. Am J Ophthalmol.

[20] Grieshaber MC, Flammer J (2007). Does the blood-brain barrier play a role in Glaucoma?. Surv Ophthalmol.

[21] Kaur C, Foulds WS, Ling EA (2008). Blood-retinal barrier in hypoxic ischaemic conditions: basic concepts, clinical features and management. Prog Retin Eye Res.

[22] Park SC (2013). Factors associated with focal lamina cribrosa defects in glaucoma. Invest Ophthalmol Vis Sci.

[23] Yamazaki Y, Koide C, Miyazawa T, Kuwagaki N, Yamada H (1991). Comparison of retinal nerve-fiber layer in high- and normal-tension glaucoma. Graefes Arch Clin Exp Ophthalmol.

[24] Kubota T (1999). Comparative study of retinal nerve fiber layer damage in Japanese patients with normal- and high-tension glaucoma. J Glaucoma.

[25] Faridi O, Park SC, Liebmann JM, Ritch R (2012). Glaucoma and obstructive sleep apnoea syndrome. Clinical & experimental ophthalmology.

[26] Liu S, Lin Y, Liu X (2016). Meta-Analysis of Association of Obstructive Sleep Apnea With Glaucoma. J Glaucoma.

[27] Charlson ME (2014). Nocturnal systemic hypotension increases the risk of glaucoma progression. Ophthalmology.

[28] Graham SL, Drance SM (1999). Nocturnal hypotension: role in glaucoma progression. Surv Ophthalmol.

[29] Konieczka K (2017). Relationship between normal tension glaucoma and Flammer syndrome. Epma j.

[30] Choi YJ, Lee EJ, Kim BH, Kim TW (2014). Microstructure of the optic disc pit in open-angle glaucoma. Ophthalmology.

[31] Lee EJ, Kim TW, Kim M, Kim H (2015). Influence of lamina cribrosa thickness and depth on the rate of progressive retinal nerve fiber layer thinning. Ophthalmology.

[32] Jonas JB, Papastathopoulos KI (1996). Optic disc shape in glaucoma. Graefes Arch Clin Exp Ophthalmol.

[33] Vongphanit J, Mitchell P, Wang JJ (2002). Population prevalence of tilted optic disks and the relationship of this sign to refractive error. Am J Ophthalmol.

[34] Samarawickrama C (2011). Myopia-related optic disc and retinal changes in adolescent children from singapore. Ophthalmology.

[35] Spaide RF, Koizumi H, Pozzoni MC (2008). Enhanced depth imaging spectral-domain optical coherence tomography. Am J Ophthalmol.

[36] Lee, E. J. *et al*. Visualization of the lamina cribrosa using enhanced depth imaging spectral-domain optical coherence tomography. *Am J Ophthalmol* **152**, 87–95 e81, (2011). 10.1016/j.ajo.2011.01.024 21570046

[37] Girard MJ, Strouthidis NG, Ethier CR, Mari JM (2011). Shadow removal and contrast enhancement in optical coherence tomography images of the human optic nerve head. Invest Ophthalmol Vis Sci.

[38] Mari JM, Strouthidis NG, Park SC, Girard MJ (2013). Enhancement of lamina cribrosa visibility in optical coherence tomography images using adaptive compensation. Invest Ophthalmol Vis Sci.

[39] Kim M, Kim TW, Weinreb RN, Lee EJ (2013). Differentiation of parapapillary atrophy using spectral-domain optical coherence tomography. Ophthalmology.

[40] Lee SH, Lee EJ, Kim TW (2016). Topographic Correlation Between Juxtapapillary Choroidal Thickness and Microstructure of Parapapillary Atrophy. Ophthalmology.

[41] Reis AS (2012). Optic disc margin anatomy in patients with glaucoma and normal controls with spectral domain optical coherence tomography. Ophthalmology.

[42] Strouthidis NG (2009). Comparison of clinical and spectral domain optical coherence tomography optic disc margin anatomy. Invest Ophthalmol Vis Sci.

[43] Lee KM, Lee EJ, Kim TW (2016). Juxtapapillary choroid is thinner in normal-tension glaucoma than in healthy eyes. Acta Ophthalmol.

[44] Jonas JB (2012). Parapapillary atrophy: histological gamma zone and delta zone. PLoS One.

