# Supplementary material for: Ocular and Clinical Characteristics Associated with the Extent of Posterior Lamina Cribrosa Curve in Normal Tension Glaucoma
Source: Sci Rep. 2018 Jan 17;8:961. doi: 10.1038/s41598-018-19321-1 (PMC5772559; doi:10.1038/s41598-018-19321-1)
Supplement: Supplementary file 2 — Supplementary Information [file 41598_2018_19321_MOESM2_ESM.pdf]

Supplementary Information

Ocular and Clinical Characteristics Associated with the Extent of Posterior Lamina Cribrosa Curve in Normal Tension Glaucoma

Seung Hyen Lee,[1] Tae-Woo Kim,[2*] Eun Ji Lee,[2] Michaël J. A. Girard,[3,4] Jean Martial Mari,[5] Robert Ritch[6]

[1]Department of Ophthalmology, Bundang Jesaeng General Hospital, Daejin Medical Center, Seongnam, Korea

[2]Department of Ophthalmology, Seoul National University College of Medicine, Seoul National University Bundang Hospital, Seongnam, Korea

[3]Department of Biomedical Engineering, National University of Singapore, Singapore

[4]Singapore Eye Research Institute, Singapore National Eye Centre, Singapore

[5]Université de la Polynésie française, Tahiti, French Polynesia

[6]Einhorn Clinical Research Center, New York Eye and Ear Infirmary of Mount Siani, New York, New York, United States

*Corresponding author

Tae-Woo Kim, MD

Professor

Department of Ophthalmology, Seoul National University Bundang Hospital,

82, Gumi-ro, 173 Beon-gil, Bundang-gu, Seongnam, Gyeonggi-do 463-707,

Korea

Tel.: 82-31-787-7374, Fax: 82-31-787-4057, E-mail: twkim7@snu.ac.kr

Supplementary information file includes one video (Video S1).

Supplementary Video legends

Supplementary Video S1

Raster scans of the optic nerve head showing that each B-scan is taken at very closely spaced intervals (about 30-34 μm). Note that the lamina cribrosa (LC) curve changes gradually along the planes. The LC curves on the adjacent planes are very similar.
